# CX_3_C chemokine receptor 1 deficiency modulates microglia morphology but does not affect lesion size and short-term deficits after experimental stroke

**DOI:** 10.1186/s12868-016-0325-0

**Published:** 2017-01-06

**Authors:** Gerlinde van der Maten, Vivien Henck, Tadeusz Wieloch, Karsten Ruscher

**Affiliations:** Laboratory for Experimental Brain Research, Division of Neurosurgery, Department of Clinical Sciences, Wallenberg Neuroscience Center, Lund University, BMC A13, 22184 Lund, Sweden

## Abstract

**Background:**

The fractalkine/CX_3_C chemokine receptor 1 (CX_3_CR1) pathway has been identified to play an essential role in the chemotaxis of microglia, leukocyte trafficking and microglia/macrophage recruitment. It has also been shown to be important in the regulation of the inflammatory response in the early phase after experimental stroke. The present study was performed to investigate if CX_3_CR1 deficiency affects microglia during the first 14 days with consequences for tissue damage after experimental stroke.

**Results:**

CX3CR1 deficiency significantly increased the number of intersections of GFP positive microglia in the proximal peri-infarct area at 2, 7 and 14 days following tMCAO compared to heterozygous and wildtype littermates. In addition, the length of microglial branches increased until day 7 in CX_3_CR1 knockout mice while the presence of a functional CX3CR1 allele resulted in a gradual reduction of their length following tMCAO. After stroke, wildtype, heterozygous and CX3CR1 deficient mice did not show differences in the composite neuroscore and assessment of infarct volumes from CX3CR1 wildtype, heterozygous and deficient mice revealed no differences between the genotypes 7 and 14 days after stroke.

**Conclusion:**

Results demonstrate that CX3CR1 deficiency affects the morphology of GFP positive microglia located in the proximal peri-infarct region during the first 14 days after tMCAO. Our data also indicate that CX3CR1 deficiency does not affect definite infarct volumes. Modulation of the CX3CR1 pathway may have implication for microglia function contributing to mechanisms of tissue reorganization in the post-ischemic brain.

## Background

For development of new therapeutics in stroke, it is important to understand mechanisms of tissue reorganisation in the brain after ischemia. Based on recent studies it is clear that parallel and partially opposite processes contribute to restoration of brain function. The interaction of multiple mechanisms including neuronal plasticity, astrogliosis, cell- and angiogenesis and inflammation are important for the final outcome after stroke.

During the recovery process, the release of pro-inflammatory molecules can initiate and perpetuate the local inflammatory response in the postischemic brain [1]. Examples of these molecules are free DNA, membrane compounds, oxidized lipids and free radicals, and they can be referred to as danger associated molecules (DAMPs). DAMPs can bind to receptors on brain resident microglia and astrocytes [2]. Cytokine and chemokine release increases, as does the expression of adhesion molecules on brain resident cells and in particular endothelial cells. Circulating immune cells bind to these molecules and migrate into the ischemic territory. These cells also release neurotoxic cytokines, chemokines and free radicals, which may exacerbate the inflammatory process [3, 4]. On the other hand, inflammatory processes contribute to tissue repair. Macrophages clear up the necrotic debris, and immune cells as well as pro-inflammatory cytokines are involved in facilitating neuronal plasticity [5].

Accumulation of immune cells is a well-regulated process and even weeks after stroke, immune cells can be detected in the ischemic territory [6]. Microglial cells are resident macrophages of the brain, and play a major role in post-injury inflammation. In the healthy brain they survey the environment, and the cells are characterized by a ramified appearance [4, 7]. In different brain pathologies, however, microglial cells transform associated with the expression of different cell surface receptors, production of immune mediators, and the capacity to phagocytise. In this state, they are indistinguishable from recruited peripheral macrophages. Previous studies have demonstrated that after stroke microglia adapt a pro-inflammatory state and contribute to post-ischemic inflammation [4, 8, 9].

Fractalkine (CX_3_CL1) is a chemokine that, in the brain, is expressed mainly by neurons, although there are some studies indicating an expression by glial cells as well [10–14]. Fractalkine exists in a membrane-bound form and, after proteolytic cleavage by ADAM10 or ADAM17, in a soluble form [15, 16]. Membranous fractalkine is involved in mechanisms of cell adhesion, its soluble form can act as chemoattractant [11]. The sole receptor for fractalkine is CX_3_C chemokine receptor 1 (CX_3_CR1) expressed on different immune cell populations [12, 17–19]. Within the first hours after ischemic injury, fractalkine expression is increased in necrotic neurons in the infarct core [18]. During the first week after stroke, there is also a delayed upregulation of fractalkine in cells of the peri-infarct area [20]. CX_3_CR1 positive cells can be detected in the ischemic tissue after 24 h, and the number of CX_3_CR1 positive microglia is strongly up-regulated during the first weeks after permanent focal ischemia [20].

The present investigation was conducted to study the effect of CX_3_CR1 deficiency on definitive tissue damage in the postischemic brain after tMCAO. Based on previous studies, we hypothesized that CX_3_CR1 deficiency may contribute to the morpholgy of microglia in the proximal peri-infarct area during the first 2 weeks with possible consequences for their function and contribution to recovery of lost neurological function after stroke.

## Methods

### Animals

For the present study transgenic male C57/B6 mice that carried green fluorescent protein (GFP) under control of the CX_3_CR1-promoter (CX_3_CR1^GFP/GFP^ and CX_3_CR1^GFP/+^) and CX_3_CR1^+/+^ wildtype littermates were used (age between 10 and 16 weeks). While CX_3_CR1^GFP/GFP^ animals do not express a functional receptor [referred to as knockout (ko) mice], CX_3_CR1^GFP/+^ mice [referred to as heterozygous (hz) mice] express GFP but still have one allele for a functional CX_3_CR1 receptor. Mice were bred as CX_3_CR1^GFP/+^ mice and offspring littermates have been used for experiments. As previously described [18] no phenotypic differences have been observed between the genotypes, body weights of wildtype (CX_3_CR1^+/+^) and deficient (CX_3_CR1^GFP/GFP^) mice did not differ before surgery (wt: 24.5 ± 3.4 g; ko: 25.9 ± 3.3 g) and on day 7 after tMCAO (wt: 21.4 ± 4.1 g; ko: 21.9 ± 2.6 g). All animal procedures included in this study were approved by the Malmö/Lund ethical committee. Experiments were carried out according the ARRIVE criteria to perform animal experiments and were blinded to the experimentators performing the surgeries.

### Transient occlusion of the middle cerebral artery

Mice were anesthetized by inhalation of initially 1.5% isofluorane in O_2_:N_2_O (30:70) and sustained at 1.0% to 1.2% throughout the occlusion period (IsobaVet, Schering-Plough Animal Health, Milton Keynes, UK). Body temperature was kept at 37 °C. Regional cerebral blood flow (rCBF) was monitored through an optical fibre probe (Probe 318-I, Perimed, Järfälla, Sweden) fixed to the skull at 1 mm posterior and 3.5 mm lateral to Bregma and connected to a laser Doppler flow meter (Periflux System 5000, Perimed). A customized silicon rubber-coated monofilament (Doccol Corporation) size 6–0, diameter 0.09–0.11 mm, length 20 mm; diameter with coating 0.25 ± 0.02 mm to 0.27 ± 0.02 mm (adapted to the age and weight of the animal) was inserted into the common carotid artery and advanced into the internal carotid artery until the origin of the middle cerebral artery (MCA), given by a sudden drop in rCBF. After 45 min, the filament was withdrawn and reperfusion was observed. The animals were placed in a heating box at 37 °C overnight, to avoid post-surgical hypothermia. Physiological parameters body weight and body temperature have been monitored throughout the first week of the study. In order to analyze the inflammatory response after tMCAO, CX_3_CR1 heterozygous and knockout mice have been sacrificed on day 2, 7 or 14 after tMCAO. In total 67 mice (wt: 23; hz: 18; ko: 26) were operated. Out of those 26 mice (wt: 9; hz: 5; ko: 12) died during the first 4 days of the study and were not included in the analysis. Numbers of animals included in analyses are indicated in respective figure legends.

### Behavioral assessment

Neurological deficits were assessed by a composite neuroscore containing the following tests: (1) animal rotation (0–5 points; 0—barely able to move but if so, moving circularly, 1—big impairment, totally rotating over the body axis or with parallel dislocation, 2—big impairment, able to walk while circumscribing small circles, 3—rotated in medium to big sized circles, 4—rotates in big circles but is able to walk straight, 5—normal function); (2) paw placement test (0–5 points; 0—paw totally immobilized, hanging down, no movement, 1—paw hanging, but little movement back and forth (horizontal plane), 2—paw hanging, horizontal and vertical movement below table’s level, 3—paw hanging horizontal and vertical movement up to table’s surface level, 4—paw hanging, mobile in all directions, reaching the table’s surface, 5—normal, paw is immediately taken up to the table’s surface); (3) forelimb flexion on suspension test (0–4 points; 0—impaired paw is not moving and tightly held to the trunk, 1—impaired paw mostly held to the trunk, slightly movable, 2—impaired paw moves up and down, asymmetry to other paw, 3—slight asymmetry between the paws, both paws well movable, 4—normal, symmetric); (4) hind limb flexion response (0–4 points; 0—impaired paw is not moving and tightly held to the trunk, 1—impaired paw mostly held to the trunk, slightly movable, 2—impaired paw moves up and down, asymmetry to other paw, 3—slight asymmetry between the paws, both paws well movable, 4—normal, symmetric) and (5) lateral pulse test (0–4 points; 0—severely impaired and asymmetrical, 1—impaired, distinct asymmetry, 2—partially impaired, visible asymmetry in response to pulse, 3—slightly impaired, almost no asymmetry, 4—normal). Animals have been tested on day 1 after the surgery and the maximum score to achieve was 22 points [20].

### Immunohistochemistry

Brain sections (thickness 30 µm) from 4% paraformaldehyde-perfused animals were rinsed in phosphate buffered saline (PBS). Endogenous peroxidase activity was quenched by washing the sections in a solution of PBS with 3% H_2_O_2_ and 10% methanol for 15 min. After subsequent rinsing in PBS, blocking was achieved by incubation in a solution containing 5% normal donkey serum (NDS) in 0.25% Triton X-100 in PBS (Tx/PBS) for 1 h. Thereafter, sections were incubated with a mouse anti neuronal nuclei (NeuN) antibody (diluted at 1:1000, Merck Millipore, Billerica, MA, USA) at 4 °C in 5% NDS in Tx/PBS overnight. The next day, sections were rinsed in 1% NDS in Tx/PBS followed by incubation with respective biotinylated secondary antibodies in 2% NDS in Tx/PBS at room temperature for 90 min. Sections were washed with Tx/PBS and incubated with avidin–biotin-complex (ABC) (Vector Laboratories, CA, USA) for 1 h. Following this incubation, the sections were rinsed in PBS. The ABC reaction was visualized using NiDAB (DAB (Dabsafe, Saveen Werner AB, Sweden)) with the addition of NiCl_2_ and a mixture of 3% H_2_O_2_ in H_2_O. Thereafter, sections were rinsed in PBS, mounted on glass slides, dried overnight, dehydrated, treated with xylene and then cover-slipped with Pertex. (Histolab AB, Sweden).

### Immunofluorescence

Brain sections (thickness 30 µm) from 4% paraformaldehyde-perfused animals were washed in PBS (3 × 10 min), blocking was achieved by 5% normal donkey serum in Tx/PBS for 60 min. The sections were then incubated with primary antibody (neuronal nuclei (NeuN), Merck Millipore, Europe; diluted at 1:1000 (ms antibody) or 1:5000 (rb antibody)) in Tx/PBS in 5% NDS at 4 °C overnight. The next day, the sections were rinsed with 1% NDS in Tx/PBS thrice for 10 min. All following steps have been performed in darkness in order to preserve the fluorophore conjugated to the secondary antibody. Sections were incubated with the secondary antibodies conjugated either with biotin or Cy3 (each diluted at 1:400) in 2% NDS in Tx/PBS for 90 min at room temperature and rinsed in PBS thrice for 10 min. Thereafter, sections incubated with biotinylated secondary antibody have been incubated with AF488 conjugated streptavidin (1:400) for 60 min at room temperature. Finally, the sections were rinsed in PBS thrice for 10 min and mounted on supercharged glass slides (Menzel, Braunschweig, Germany), allowed to dry, cover-slipped using PVA-DABCO (Saveen-Werner, Malmö, Sweden) and analysed using LSM 510 confocal microscopy (Zeiss Jena, Germany).

### Infarct size measurements

Brains from perfusion fixed animals were cut into coronal sections with a thickness of 30 µm and stained for neuronal nuclei (NeuN) [20]. The noninjured portion of the ipsilateral and contralateral hemisphere were encircled and the indirect infarct volume was calculated by integration of areas from serial sections of each brain as described previously [20].

### Sholl analysis

**Fig. 1:**
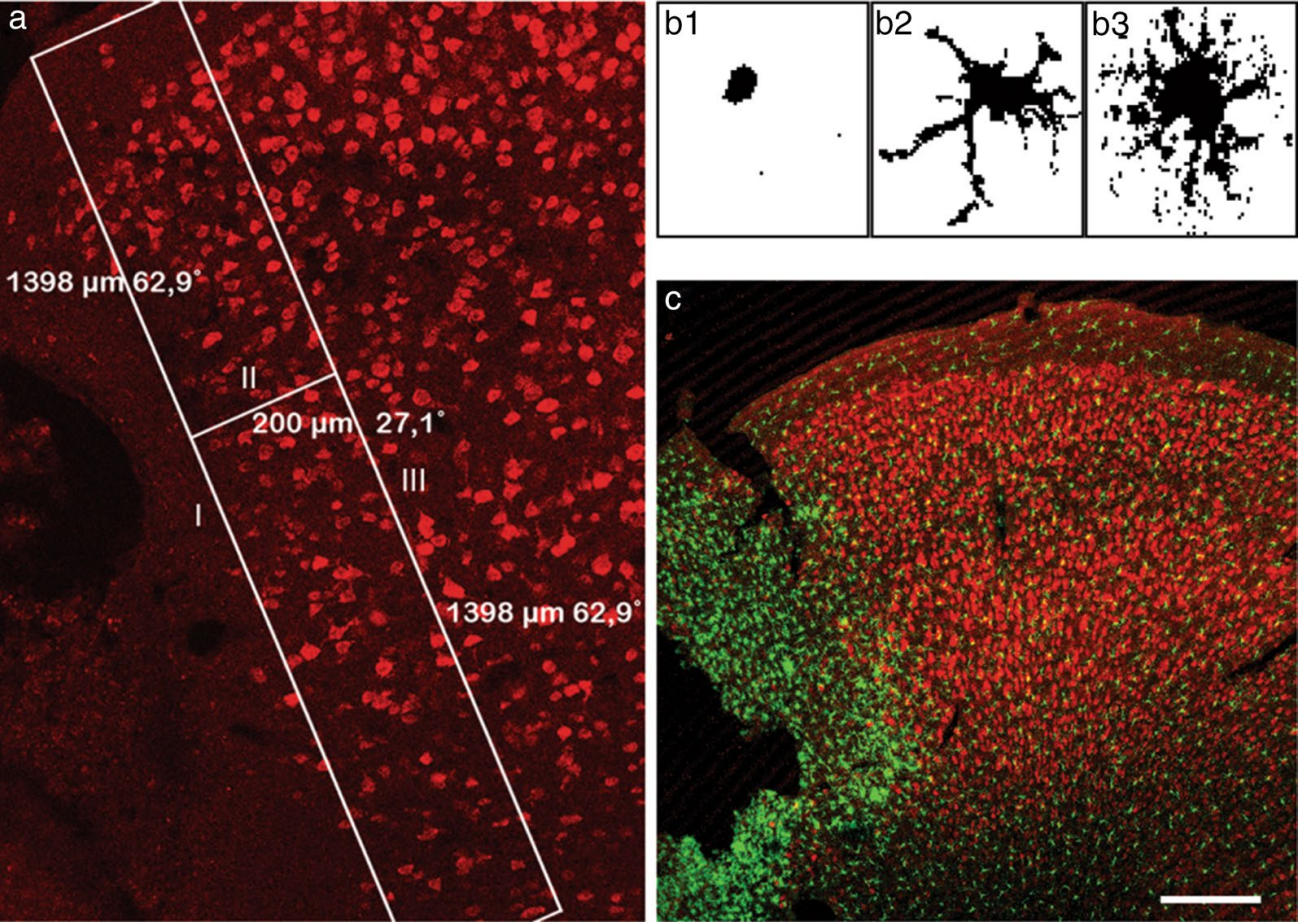
Definition of the region of interest for Sholl analysis. **a** Regions of interest for cell analysis and different morphologies of GFP^+^ cells. **b1** amoeboid cell, **b2** intermediate ramified cell, **b3** ramified cell. **c** Superimposed micrograph showing NeuN (Cy3, *red*) and GFP^+^ cells in the ischemic hemisphere, the infarct core and adjacent peri-infarct area, *scale bar* 200 µm

Sholl analysis determines the number of intersections of a cell with a subset of circles, with increasing diameters starting from the centre of the cell (Fig. 1) [21]. This gives an indication of the degree of ramification of a cell. Z-stacked micrographs from coronal brain sections of CX_3_CR1^+/+^, CX_3_CR1^GFP/+^ and CX_3_CR1^GFP/GFP^ mice (3 sections per animals from the following levels related to bregma: 1.2–1.0; 0; −1.0 to −1.2) stained for NeuN were acquired using a Zeiss LSM510 laser scanning confocal microscope and merged into a single plane image using the LSM Image Browser software. Based on the NeuN staining the peri-infarct area was defined as follows: the NeuN staining was used to delineate the infarct border with a line (line I). From this line, a 200 µm long line was drawn at 90 degrees (line II). Parallel to line I, a line (line III) was drawn at the endpoint of line II to mark the distal border of the peri-infarct area. Using these coordinates, the rectangular area was used for analysis of Iba-1^+^ or GFP^+^ cells (Fig. 1a). Per picture, randomly 50 cells were selected in the peri-infarct area, using a circle with a circumference of 140 µm (140/π in diameter). The following selection criteria for analysis were defined: (1) a cell needed to fit in the circle completely; (2) a cell needed to have a visible soma and (3) a cell should not overlap with other cells. The extracted cells were saved in separate TIFF files. Using ImageJ pictures were converted to 8-bit files with a set threshold [22]. The center of a cell was defined manually, and Sholl analysis was performed with a starting radius of 3 pixels and steps of 1 pixel. Starting with a circle with a diameter of 3 pixels and expanding the circles with 1 pixel every time, intersections per circle were counted using the ImageJ Sholl analysis plugin. Results from each cell per conditions were integrated and analyzed using Microsoft Excel. Analysis has been performed blinded to the examiner.

### Statistical analysis

Statistical analysis was performed using Excel and IBM-SPSS (Version 22, IBM, Stockholm, Sweden). Sholl analysis data are presented as medians with the 25th and 75th percentiles. Comparison between groups was performed using the Student’s *t* test for GFP positive cells and Kruskal–Wallis analysis for Iba-1 positive cells. Infarct volumes are presented as mean ± standard deviation (SD). Statistical analysis has been performed by one-way analysis of variance. No statistical difference has been reached at 7 days (p = 0.3049) and 14 days (p = 0.6128), posthoc corrections were not performed. Neuroscore data are presented as medians with the 25th and 75th percentiles and whiskers. Statistical analysis have been performed by the Kruskal–Wallis test (p = 0.8196).

## Results

### Effect of CX_3_CR1 deficiency on the morphology of microglia after stroke

**Fig. 2:**
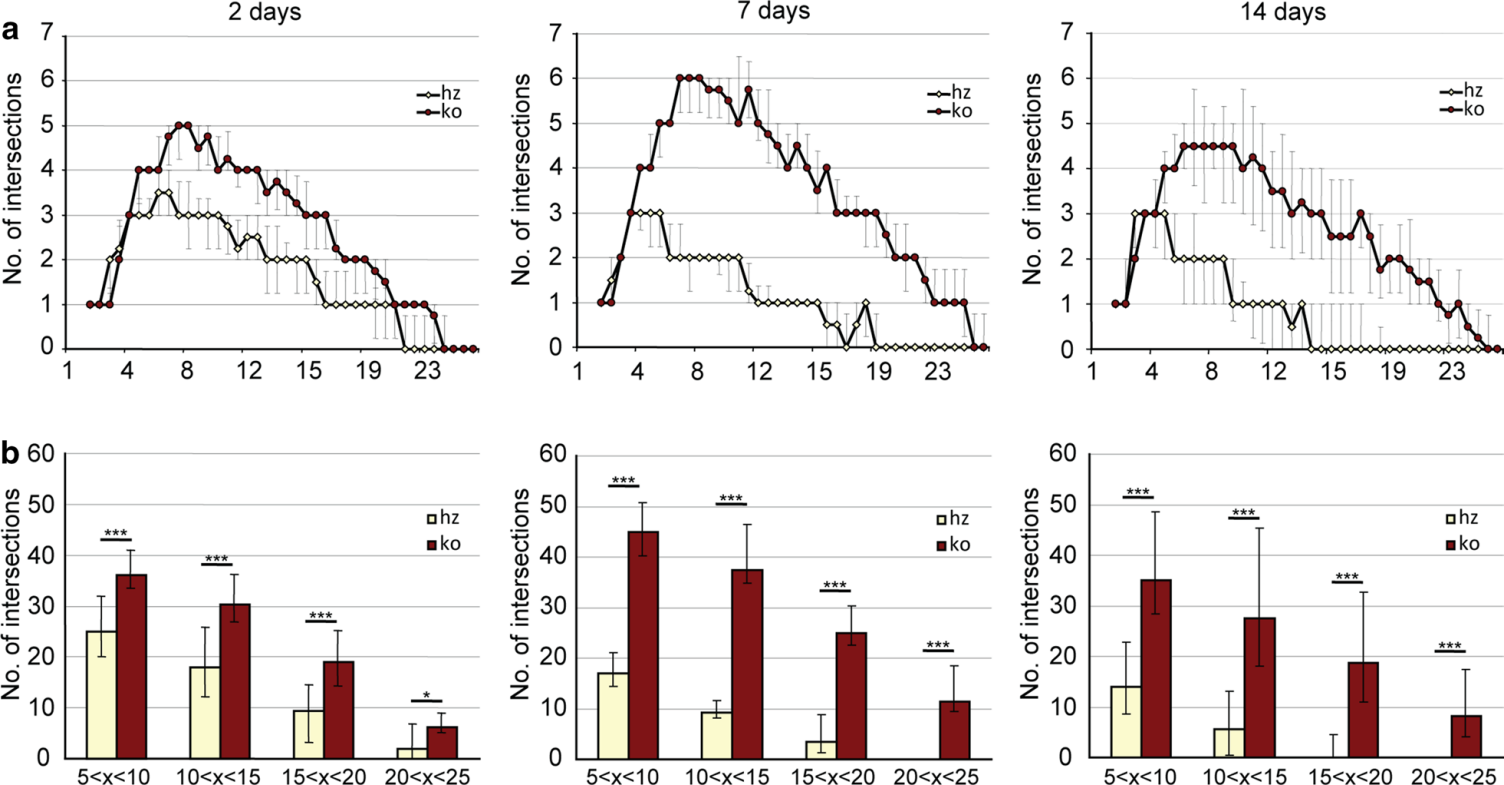
Sholl analysis after transient occlusion of the middle cerebral artery. **a** Distribution of intersections in heterozygous and knockout mice at 48 h, 7 and 14 days after tMCAO. Data are presented as medians with the 25th and 75th percentiles. In total 3 sections per animal were analyzed of animals: hz 48 h = 2, hz 7 days = 2, 14 days hz = 3, ko 48 h = 3, ko 7 days = 3, ko 14 days = 3. **b** Number of crossing in the indicated intervals in heterozygous and knockout mice at 48 h, 7 and 14 days after tMCAO. Data are presented as medians with the 25th and 75th percentile. Statistical analysis has been performed by using the Student’s *t* test for individual interval groups: *p < 0.05, ***p < 0.001

**Fig. 3:**
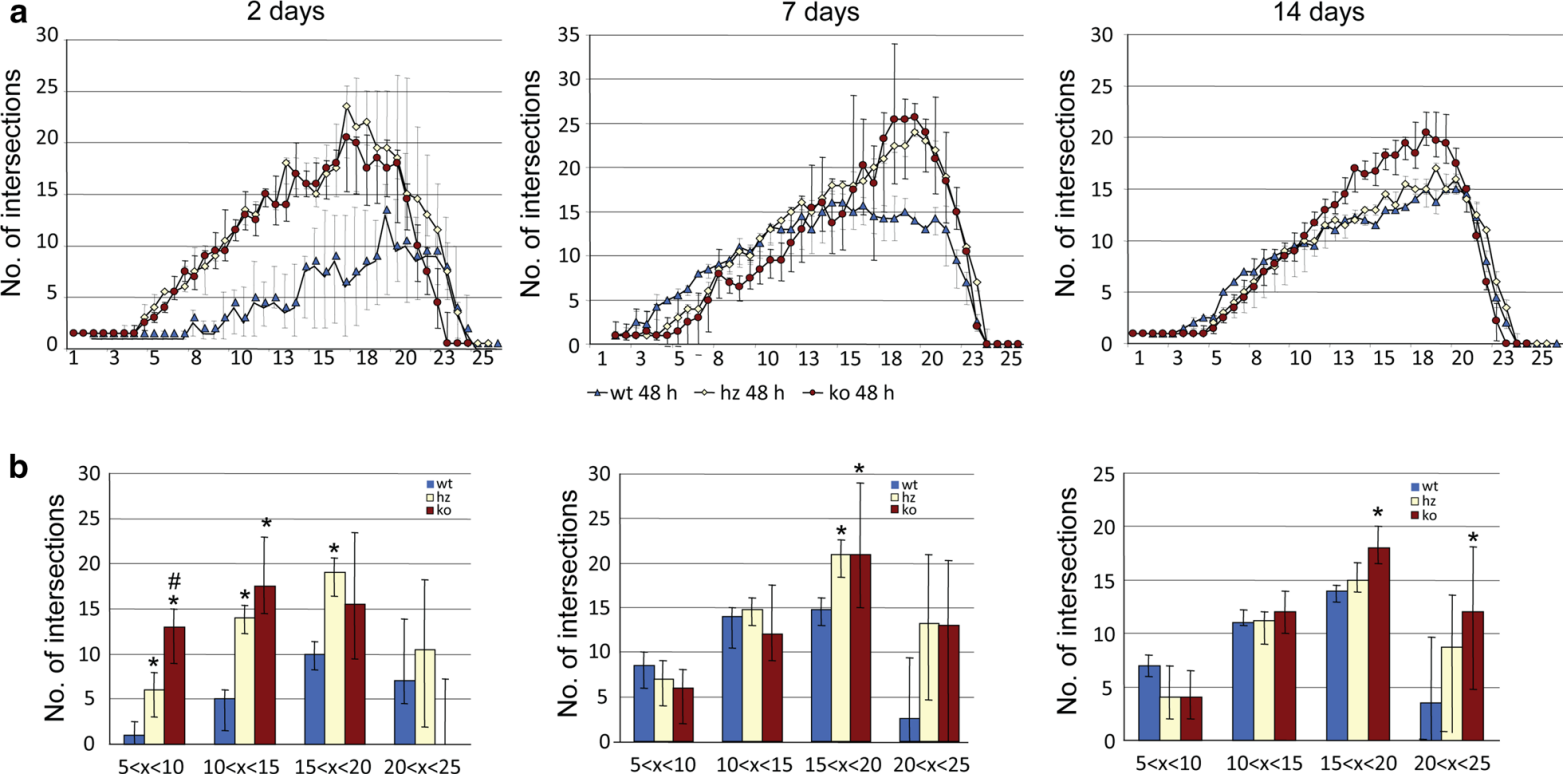
Sholl analysis of Iba-1 positive microglia in the proximal peri-infarct area. **a** Distribution of intersections in wildtype, heterozygous and knockout mice at 48 h, 7 and 14 days after tMCAO. Data are presented as medians with the 25th and 75th percentiles. In total 3 sections per animal were analyzed of animals: wt 48 h = 3, wt 7 days = 4, 14 days wt = 4, hz 48 h = 2, hz 7 days = 3, 14 days hz = 3, ko 48 h = 3, ko 7 days = 3, ko 14 days = 6. **b** Number of crossing in the indicated intervals in heterozygous and knockout mice at 48 h, 7 and 14 days after tMCAO. Data are presented as medians with the 25th and 75th percentile. Statistical analysis has been performed by Kruskal–Wallis: *p < 0.05 versus wt, ^#^p < 0.05 versus hz

**Fig. 4:**
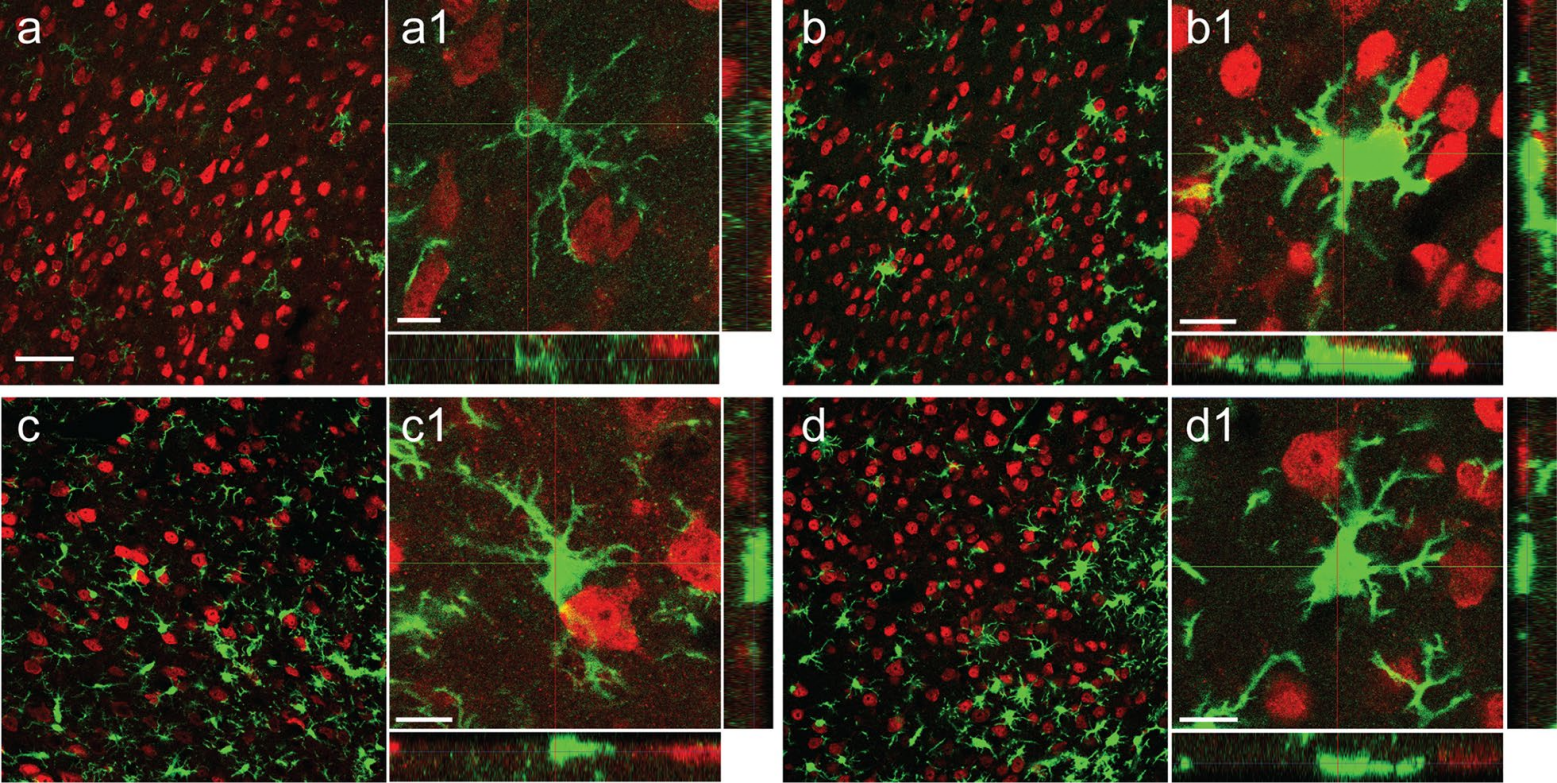
Microglia morphology after transient occlusion of the middle cerebral artery. Immunofluorescence analysis of Iba-1 positive cells (AF488, *green*) in a wildtype mouse (**a**, higher magnification in **a1**) and CX_3_CR1 deficient mouse (**b**, higher magnification in **b1**) on day 2 and in a wildtype mouse (**c**, higher magnification in **c1**) and CX_3_CR1 deficient mouse (**d**, higher magnification in **d1**) on day 14 (**c**, higher magnification in **c1**) following PT. Sections were costained with NeuN (Cy3, *red*). *Scale bars* **a**–**d** 50 µm; **a1**–**d1** 10 µm

Previous studies provided data showed that CX_3_CR1 deficiency affects the morphology of immune cells in the ischemic territory during the first 24 h after stroke [23]. We hypothesized that microglia morphology indicative for their activation status is affected by the presence of CX3CR1 beyond the time window of neuroprotection after stroke. An established Sholl analysis protocol has been applied to study the morphology of GFP^+^ cells in the peri-infarct area of CX_3_CR1^GFP/+^ (heterozygous) and CX_3_CR1^GFP/GFP^ (knockout) mice at 48 h, 7 and 14 days after transient occlusion of the middle cerebral artery (tMCAO) (Fig. 1). Coronal sections were stained for the neuronal marker NeuN in order to exactly delineate the infarct border. As shown in Fig. 2, GFP^+^ cells from heterozygous mice displayed less intersections compared to GFP^+^ cells in knockout mice at all three time points. In addition, the number of intersections decreased over time in heterozygous mice. In contrast, ramification of GFP^+^ microglia in knockout mice further increased until day 7 after tMCAO and only declined 14 days following tMCAO to levels found on day 2. To assess the data for statistical analysis, intersections were grouped depending on distance from the center of a cell (5–10, 10–15, 15–20 and 20–25 µm). As shown in Fig. 2b, a significant higher number of interscetions was observed in CX_3_CR1 deficient mice compared to heterozygous littermates at all time points and in all groups after tMCAO. Likewise, Sholl analysis from Iba-1 positive cells showed a significant higher number of intersections in CX3CR1^GFP/+^ and CX3CR1^GFP/GFP^ mice at 48 h (Fig. 3). In contrast, to the analysis based on GFP positive cells analysis of intersections showed no differences in intersections between CX3CR1^GFP/+^ and CX3CR1^GFP/GFP^ mice at 48 h and 7 days following PT. At 14 days after PT a reduction of interscections was observed in CX3CR1^GFP/+^ mice compared to knockout littermates. The number was similar to the number found in wildtype mice, however, did not reach statistical significance. Results are supported by morphology of Iba-1 positive microglia cells in the proximal peri-infarct area (Fig. 4). While microglia cells in wildtype animals displayed a more stellate morphology, cells found in knockout mice appeared with bigger soma and thicker branches (Fig. 4a, b). On day 14, morphology appeared more equalized, however, with a lower number of intersections in wildtype microglia (Fig. 4c, d). From this analysis we conclude that CX_3_CR1 deficiency significantly affects ramification of microglia cells in the proximal peri-infarct area after tMCAO. The level of ramification might be indicative for a different status of cell activation in animals expressing a functional CX_3_CR1 receptor.

### Impact of CX3CR1 deficiency on infarct volume and functional outcome after tMCAO

**Fig. 5:**
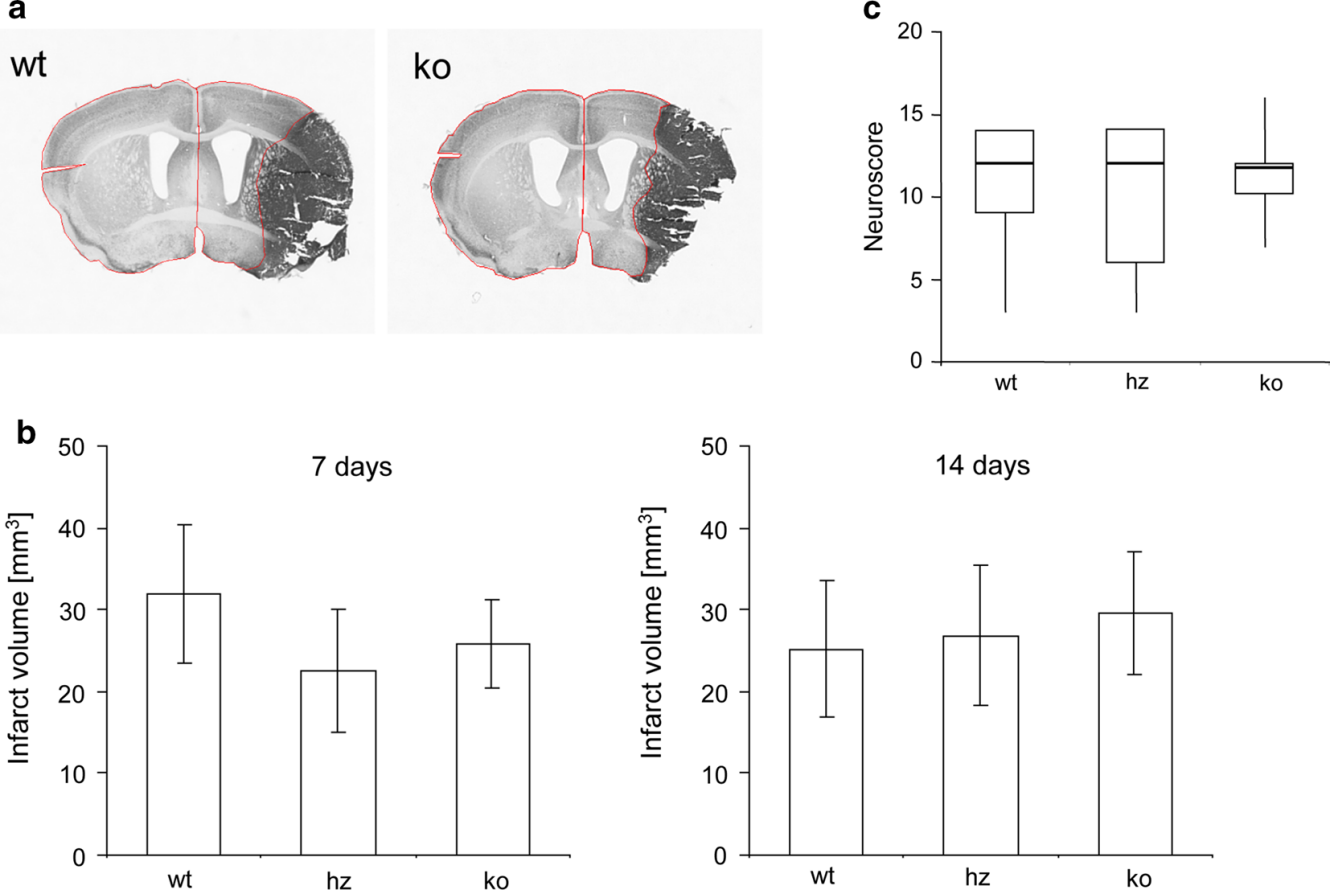
Functional outcome and lesion volumes after tMCAO. **a** Representative coronal sections from a wildtype (wt) and a CX_3_CR1 deficient (ko) mouse at day 7 following tMCAO. The encircled brain regions (contralateral hemisphere and non-injured part of the ischemic hemisphere) were used for lesion volume calculations. **b** Infarct volumes on day 7 and day 14 in wildtype (wt), heterozygous (hz) and CX_3_CR1 deficient mice (ko). Data are presented as mean ± SD. One-way analysis of variance. No statistical difference has been obtained at 7 days (p = 0.30) and 14 days (p = 0.61), respectively. **c** Composite neuroscore obtained on day 1 after tMCAO from wildtype (wt; n = 12), heterozygous (hz; n = 11) and CX_3_CR1 deficient mice (ko; n = 14). Scores are presented as medians with the 1st and 3rd *quartile* and *whiskers*. No statistical difference was found between the experimental groups (Kruskal–Wallis, p = 0.82)

Seven and 14 days after tMCAO, mice were perfusion fixed and series of coronal sections with defined distances were stained for the neuron specific marker NeuN. Based on NeuN immunoreactivity the noninjured portion of the ischemic hemisphere and the contralateral hemisphere were encircled and infarct volumes were calculated as described previously (Fig. 5a) [24]. As shown in Fig. 5b, infarct volumes did not differ between the wildtype (33.23 ± 6.5 mm^3^, n = 5), heterozygous (22.5 ± 7.5 mm^3^, n = 5) and CX_3_CR1 deficient mice (29.84 ± 7.5 mm^3^, n = 5) on day 7 and day 14 (wt: n = 6, 25.32 ± 8.9 mm^3^; hz: n = 3, 26.9 ± 8.5 mm^3^; ko: n = 6, 27.78 ± 9.2 mm^3^). Likewise, no differences in the neurological tests have been obtained in wildtype (n = 12) mice compared with heterozygous (n = 11) and CX_3_CR1 deficient mice (n = 14) after tMCAO (Fig. 5c). From these experiments we conclude that CX_3_CR1 deficiency did not affect the loss immediate neurological function and also has no effect on the definitive lesion size after transient occlusion of the middle cerebral artery for 45 min (Fig. 5).

## Discussion

The present study was conducted to characterize the inflammatory response and in particular the relevance of CX_3_CR1 on microglia morpholgy and lesion size during the first 14 days after transient occlusion of the middle cerebral artery. We found that CX_3_CR1 deficiency significantly altered the microglial response in the proximal peri-infarct area. We also provide the evidence that CX_3_CR1 knockout per se has no effect on lesion size and early functional outcome after tMCAO. We will discuss the relevance of CX_3_CR1 activation for immune cell activation after stroke. Moreover, we will elaborate on the consequences of CX_3_CR1 knockout on different results obtained in previous studies in regard to functional outcome and lesion size after experimental stroke.

### Relevance of CX_3_CR1 for immune cell activation after stroke

It has been shown that activation of the CX_3_CR1 receptor is involved in microglia/leukocyte trafficking [10, 16, 19, 25, 26] and phagocytosis [23, 27]. These processes are associated with changes in cell morphology. In the present study Sholl analysis has been performed to test if changes in ramifications in GFP^+^ cells depend on a functional CX_3_CR1 receptor in the peri-infarct area after tMCAO. We observed a significant higher number of ramifications in CX_3_CR1 deficient mice compared to heterozygous littermates at 2, 7, and 14 days after tMCAO. Similar results have been obtained 24 h after tMCAO [23]. In addition, we performed Sholl analysis based on Iba-1 immunoreactivity. Also here, a significant higher number of intersections was observed in CX_3_CR1 deficient mice. These results might be indicative for a function of the CX_3_CR1 in regard to morphological changes of brain resident microglia in the postischemic hemisphere. Throughout the observation period of 14 days after tMCAO, a continuous reduction of intersections and a reduction in length of microglial branches were found in mice with a functional CX_3_CR1 allele. In contrast, an increase of intersections (GFP positive cells) or higher number of intersections at all time points (Iba-1 positive cells) were observed in CX_3_CR1 deficient mice during the first week after tMCAO with the biggest difference between the genotypes during the first week after tMCAO. Described morphological changes between the genotypes might be linked to changes of microglia function, such as phagocytosis, as shown previously [20, 23, 27]. Our results may also support earlier studies describing reduced phagocytosis in cultivated wildtype microglia cell treated with fractalkine [27]. Differences in microglia morphology between the genotypes might also be explained by their susceptibility to the endogenous CX3CR1 ligand fractalkine. We have shown that AMD3100, an irreversible antagonist on the CXCR4 and partial agonist on the CXCR7, reduced the level of fractalkine in CX3CR1 heterozygous mice. Treatment is associated with a reduced number of microglia and a deduction of their soma size in the proximal peri-infarct area [20]. Together, results suggest that the dynamics of microglia morphology and activation are a very fine tuned processes. Already slightly elevated levels of fractalkine found in wildtype and heterozygous mice but also the lack of CX3CR1 and downstream signaling cascades may have consequences for microglia function such as the release of pro-inflammatory molecules [20].

### The role of the fractalkine/CX3CR1 pathway for functional outcome and tissue damage after stroke

To reiterate, initial studies have demonstrated that the fractalkine/CX_3_CR1 pathway is instrumental in chemotaxis of microglia [12, 25], leukocyte trafficking and microglia/macrophage recruitment [16]. In in vitro models fractalkine showed neuroprotective properties [28] by activation of neuronal cell survival pathways [29], and fractalkine also led to increased survival of microglia [30]. In addition, CX_3_CR1 deficient mice subjected to transient occlusion of the middle cerebral artery (tMCAO) showed smaller infarct volumes compared to heterozygous and wildtype littermates analyzed 72 h after tMCAO. CX3CR1 deficient animals also showed reduced levels of pro-inflammatory cytokines. Reduced levels of pro-inflammatory cytokines and reduced numbers of accumulating immune cells, however, might have been the result of smaller infarcts [31, 32]. Findings have been confirmed in a model of permanent MCAO (pMCAO) [27]. Moreover, administration of fractalkine to wildtype mice also resulted in smaller infarcts suggesting neuroprotective actions of the fractalkine/CX3CR1 pathway. In contrast, administration of fractalkine to fractalkine deficient mice resulted in bigger lesions. Summarizing both studies, administration of fractalkine but also CX_3_CR1 deficiency seem to be beneficial after experimental stroke and models of neurodegeneration [19]. We, therefore, aimed at studying the effect of CX3CR1 deficiency on acute functional deficits and definite lesion size. As described above, no differences have been observed between CX3CR1 deficient mice and wildtype littermates. Results are based on a sufficient number of animals included in the studies, physiological parameters have been controlled tightly and mortality was not different between the study groups. Infarct size measurements have been performed based on NeuN immunoreactivity, and respective areas (see “Methods” section) have been encircled with the aid of microscopic control. In our approach we applied a commonly used mouse anti NeuN antibody, hence it needs to be considered that subsequent application of secondary anti mouse antibodies resulted in crossreactivity with extravasated IgG’s in the ischemic core. This can be eliminated by the use of mouse anti NeuN antibodies directly conjugated with biotin or anti NeuN antibodies from other species. However, detection of IgG extravasation can facilitate distinction between intact and ischemic brain tissue. Shrinkage of tissue has been eliminated by indirect infarct size measurements as described previously [24]. The approach to stain postischemic brains (TTC versus NeuN immunohistochemistry) as well as different time points of infarct size measurements may explain the differences between the studies. Although different methods are available to evaluate the size of brain lesions after experimental stroke [33] our data suggest that results from low resolution methods such as TTC or MRI should be verified by immunohistochemistry. In addition, analysis of ischemic lesions should be performed at a time point when the infarct has been subsided and edema has been resolved. Since differences are apparent between experimental stroke models and investigators, analysis should not be performed before day 7 after stroke. From our studies we conclude that CX3CR1 deficiency has no impact on acute functional outcome and did not affect lesion size after tMCAO.

## Conclusion

We show that CX3CR1 significantly contributes to changes in microglia morphology in the proximal peri-infarct area following tMCAO. Morphological alterations suggest a shift in microglia functionality integrated in the inflammatory response after stroke. We also demonstrate that CX3CR1 deficiency has no beneficial effect on lesion size neither it affects early functional outcome after stroke. This, in first line, can be explained by similar lesion sizes in mice of the different genetic backgrounds. Changes in the number of branches might have consequences in the interaction of microglia with other glial cell populations and neurons. Therefore, modulation of microglia in morphology and function, e.g. by pharmacological means, might be a therapeutic strategy to optimize tissue reorganization in the post-ischemic brain.
